# How the *in situ* monitoring of bulk crystalline phases during catalyst activation results in a better understanding of heterogeneous catalysis

**DOI:** 10.1039/d1ce00817j

**Published:** 2021-09-01

**Authors:** Simon Penner

**Affiliations:** Institute of Physical Chemistry, University of Innsbruck Innrain 52c A-6020 Innsbruck Austria simon.penner@uibk.ac.at +4351250758003

## Abstract

The present Highlight article shows the importance of the *in situ* monitoring of bulk crystalline compounds for a more thorough understanding of heterogeneous catalysts at the intersection of catalysis, materials science, crystallography and inorganic chemistry. Although catalytic action is widely regarded as a purely surface-bound phenomenon, there is increasing evidence that bulk processes can detrimentally or beneficially influence the catalytic properties of various material classes. Such bulk processes include polymorphic transformations, formation of oxygen-deficient structures, transient phases and the formation of a metal–oxide composite. The monitoring of these processes and the subsequent establishment of structure–property relationships are most effective if carried out *in situ* under real operation conditions. By focusing on synchrotron-based *in situ* X-ray diffraction as the perfect tool to follow the evolution of crystalline species, we exemplify the strength of the concept with five examples from various areas of catalytic research. As catalyst activation studies are increasingly becoming a hot topic in heterogeneous catalysis, the (self-)activation of oxide- and intermetallic compound-based materials during methanol steam and methane dry reforming is highlighted. The perovskite LaNiO_3_ is selected as an example to show the complex structural dynamics before and during methane dry reforming, which is only revealed upon monitoring all intermediate crystalline species in the transformation from LaNiO_3_ into Ni/La_2_O_3_/La_2_O_2_CO_3_. ZrO_2_-based materials form the second group, indicating the *in situ* decomposition of the intermetallic compound Cu_51_Zr_14_ into an epitaxially stabilized Cu/tetragonal ZrO_2_ composite during methanol steam reforming, the stability of a ZrO_0.31_C_0.69_ oxycarbide and the gas-phase dependence of the tetragonal-to-monoclinic ZrO_2_ polymorphic transformation. The latter is the key parameter to the catalytic understanding of ZrO_2_ and is only appreciated in full detail once it is possible to follow the individual steps of the transformation between the crystalline polymorphic structures. A selected example is devoted to how the monitoring of crystalline reactive carbon during methane dry reforming operation aids in the mechanistic understanding of a Ni/MnO catalyst. The most important aspect is the strict use of *in situ* monitoring of the structural changes occurring during (self-)activation to establish meaningful structure–property relationships allowing conclusions beyond isolated surface chemical aspects.

## Abstract 2

This Highlight article shows the importance of the *in situ* monitoring of bulk crystalline compounds for a thorough understanding of heterogeneous catalysts at the intersection of catalysis, materials science, crystallography and inorganic chemistry.

## Graphical abstract

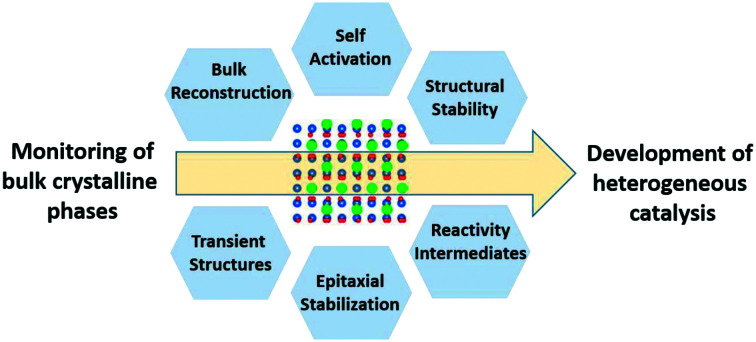

## 1. Introduction into the concept

The central goal in heterogeneous catalytic research is the determination of and knowledge about catalytically active sites.^1^ Characterization of active sites is hampered by the existence of the so-called “pressure” and “materials” gaps.^2^ These are related to the general inability to directly transfer the characterization under ultra-high vacuum conditions to technical conditions at elevated pressures and to derive mechanistic ideas from idealized model systems for usually ill-defined technical powder catalysts. *In situ* or *operando* characterization is therefore necessary to eventually monitor the formation and catalytic action of active sites.^3^ The last decades have seen a tremendous rise in the development of such structural and spectroscopic characterization methods, and to date, catalyst characterization under real operating conditions has been possible using a plethora of experimental methods.^3^ These include, but are not limited to, classical catalyst characterization tools like electron microscopy, X-ray photoelectron spectroscopy, infrared spectroscopy and X-ray diffraction.^3^ Although catalysis is an almost exclusively surface-bound phenomenon, bulk *in situ* characterization tools have contributed greatly to the thorough understanding of catalytic action. Morphological changes of metal particles in reactive gas atmospheres,^4^ gas-phase induced polymorphic transformations of oxides^5^ and reaction-induced exsolution of metal particles from perovskites^6^ are all bulk-related phenomena that have a direct catalytic consequence. As the most powerful structural characterization tool, X-ray diffraction is able to deliver qualitative and quantitative information on the structural and crystallographic properties of mostly bulk crystalline species of a catalyst. If it is carried out under *in situ* reaction conditions, it allows the monitoring of the development of crystalline species as a function of temperature and reaction gas environment and, by correlation with catalytic profiles, also the establishment of structure–activity correlations.^7^ It is worth noting that the *in situ* monitoring allows the potential influence of transient crystalline species on the catalytic properties to be unraveled, which cannot inherently be studied if *ex situ* characterization of the spent catalyst state is carried out. This phenomenon is particularly pronounced if the active phase is formed *via* a pathway of self-activation, *e.g.*, as observed in the decomposition of intermetallic compounds^8^ or the exsolution of metallic particles from perovskite materials.^9,10^

In this Highlight article we provide an introduction to the widescale possibilities of monitoring the *in situ* development of crystalline (transient) species through pre-treatments and self-activation to derive a better mechanistic understanding of heterogeneous catalysts and their constituting entities. To generalize the concept, we focus on a set of case studies spanning materials from oxide and oxy-carbides over complex oxides to intermetallic compounds in a number of important reactions, where the monitoring of crystalline species contributes to a thorough understanding. These reactions are methanol steam reforming (MSR) and dry reforming of methane (DRM).

The present Highlight article attempts to raise awareness of the importance of the development and evolution of bulk crystalline species. The impact of especially bulk species on catalysis is very common and widespread, and we show in selected examples how this influence manifests itself in various ways and what we can learn from suitable monitoring of bulk species to derive mechanistic principles and catalyst synthesis strategies. We emphasize here that the impact of the bulk species refers to features that can be directly correlated to activity changes. Of course, these changes have direct consequences for the surface structure of the catalysts that dominate the adsorption and reactivity of reactive species. Naturally, this approach is particularly worthwhile when crystalline species are present and can be studied by the full artillery of *in situ* bulk structural characterization methods, with *in situ* X-ray diffraction at the forefront. Hence, the topic of this Highlight article is centered at the intersection of crystal engineering, inorganic chemistry and catalysis. Disentangling the different influences is therefore imperative, and the selected examples will provide evidence of how catalyst synthesis (*i.e.*, inorganic chemistry), crystal engineering/crystallography (*i.e.*, steering the crystal modification of the catalyst material by, *e.g.*, annealing treatments in different gas atmospheres) and catalysis (*i.e.*, the catalytic characterization) always work together to derive general principles.

To show the general validity of the concept, we group the example materials into two classes: ZrO_2_-based materials^5,8^ and general metal–oxide systems.^9–11^ The latter are studied to highlight the beneficial properties of a crystalline metal–oxide phase boundary, either formed by *in situ* activation or by a co-precipitation approach. The respective samples are (i) a LaNiO_3_ perovskite that is *in situ* decomposed into Ni/La_2_O_3_/La_2_O_2_CO_3_ (ref. 9 and 10) and (ii) a 5% Ni/MnO catalyst.^11^ The recurrent theme is the importance of the *in situ* monitoring of bulk crystalline phases to establish valid structure–activity correlations. While for LaNiO_3_ the proper knowledge of the sequence of structural transformations is important, the 5% Ni/MnO catalyst is used to highlight the importance of monitoring the crystalline bulk carbon evolution during DRM to infer pathways of beneficial carbon clean off reactivity. The second materials group is centered around ZrO_2_, and in three examples, the importance of the proper understanding of the crystalline bulk reactivity is put forward. The three examples comprise the *in situ* decomposition of a Cu_51_Zr_14_ intermetallic compound during methanol steam reforming,^8^ the *in situ* oxidation/decomposition of a Zr-oxycarbide material for electrochemical applications^12^ and a general discussion about the complexity of the crystalline Zr–O phase diagram, manifesting itself in a complex gas-phase dependence of the tetragonal-to-monoclinic ZrO_2_ structure transformation.^5^ The latter has tremendous influence on all catalytic applications of ZrO_2_, and the understanding of the evolution of the bulk crystalline phases is the key to the mechanistic understanding. Despite the obviously different chemical natures of the materials, the examples are carefully selected to emphasize the common links and leading themes that can thus be transformed from one sample to another and allows projections of reactivity along the material axis. For each example discussed in section 2, a short introduction to the state-of-the-art is given to emphasize the existing problems and, in turn, how the proposed monitoring of the evolution of bulk crystalline structures can contribute in finding associated solutions.

## 2. Detailing the concept by discussion of selected examples

### 2.1. *In situ* activation of DRM catalysts: LaNiO_3_

**Fig. 1:**
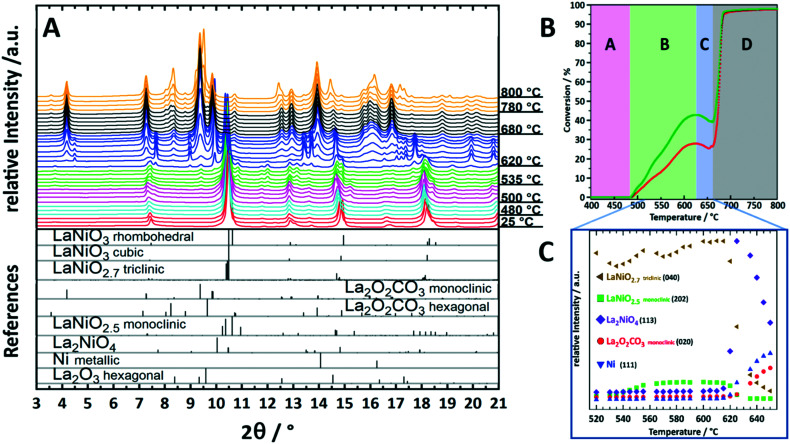
Characterization and structure–activity correlation during decomposition of crystalline LaNiO_3_ in a 1 : 1 CO_2_ : CH_4_ dry reforming mixture up to 800 °C. Panel A: Synchrotron-based *in situ* X-ray diffractograms during DRM operation. The diffractograms with similar structures are colored accordingly. The reference patterns are shown as bars at the bottom of the panel. Panel B: Catalytic DRM profile of LaNiO_3_. The sections from A–D indicate temperature regions, where important structural alterations are present.^9^ Panel C: Semi-quantitative analysis by the Le Bail method of the peak intensities of important structures in the temperature region 520–660 °C as discussed in ref. 9. To facilitate the correlation between the DRM activity and structural evolution in panel C, the temperature region is marked in panel B on the temperature axis. Reproduced with permission from ref. 9. Copyright American Chemical Society, 2021.

To illustrate the importance of monitoring transient crystalline species during catalyst activation, we will focus on the behavior of the archetypical perovskite material LaNiO_3_ in methane dry reforming.^9,10^ Mostly due to their outstanding redox properties, perovskite materials with the general formula ABO_3_ have evolved as a particularly promising catalyst class for methane dry reforming.^13^ To form active phases, perovskites are used as precursor materials and are converted into active metal/oxide materials by dedicated activation treatments, either in a hydrogen atmosphere or directly in the CO_2_/CH_4_ dry reforming mixture. LaNiO_3_ is a highly active DRM pre-catalyst that can be transformed into a Ni/La_2_O_3_/La_2_O_2_CO_3_ composite by the activation treatment.^9,10^*Ex situ* characterization of the spent catalyst state in fact reveals the final structure of LaNiO_3_ after activation, but gives no answer on the individual steps of LaNiO_3_ activation and eventual differences between hydrogen or DRM self-activation. Establishment of structure–activity correlations is therefore basically excluded. Fueled by a number of studies hinting towards the importance of *in situ* monitoring of structural catalyst activation,^14,15^ we have embarked on a detailed *in situ* study of LaNiO_3_ during catalyst activation by synchrotron-based X-ray diffraction.^16,17^ Due to the high beam intensity and the fast detector read-out, we were able to deliver unprecedented insight into the structural transformations during activation and the direct relation to catalytic DRM activity. In fact, the structural transformation of LaNiO_3_ into Ni/La_2_O_3_/La_2_O_2_CO_3_ is very complex and involves a number of consecutive steps that all feature a distinct catalytic fingerprint (Fig. 1). The first transformation is a polymorphic transformation of orthorhombic LaNiO_3_ into a tetragonal structure, followed by the formation of oxygen-deficient LaNiO_2.7_ and LaNiO_2.5_. Both appear as crystalline phases (Fig. 1A) and are already associated with moderate DRM activity (Fig. 1B and C). The crucial step for strong acceleration of DRM activity is the decomposition of those sub-stoichiometric structures into the crystalline Ruddlesden–Popper structure La_2_NiO_4_ and the in-parallel exsolution of metallic Ni and formation of La_2_O_3_, which quickly carbonates to monoclinic La_2_O_2_CO_3_ (Fig. 1B and C). La_2_NiO_4_ itself is a transient crystalline structure, which decomposes at 620 °C into metallic Ni and La_2_O_3_/monoclinic La_2_O_2_CO_3_, giving rise to strong acceleration of activity. Above 680 °C, a polymorphic monoclinic-hexagonal La_2_O_2_CO_3_ transformation sets in, which is accompanied by exceeding the stability limit of the La-oxycarbonate structures beyond 770 °C, where hexagonal La_2_O_3_ is re-formed by CO_2_ release. The final structure, persisting upon re-cooling to room temperature, is Ni/La_2_O_3_/La_2_O_2_CO_3_. The latter is re-formed during the cooling process. Hence, between 500 °C and 800 °C the catalytic activity can be directly related to a set of transformations of crystalline species (Fig. 1B and C). We might even explain the dip in catalytic activity between 620 °C and 650 °C by the evolution of crystalline species: in this temperature region, La_2_NiO_4_ starts to fully decompose, releasing lattice oxygen to oxidize the exsolved metallic Ni on the surface. NiO is a poor methane activator; hence the catalytic activity decreases as long as the oxygen supply does not cease. Only above 650 °C, the decomposition of La_2_NiO_4_ is complete and no oxygen can be delivered anymore. The Ni particles, hence, remain metallic, and the DRM activity increases due to improved methane activation. By comparing the self-activation properties under DRM operation conditions to those during pre-reduction in a hydrogen atmosphere we were able to show the crucial role of the crystalline La_2_NiO_4_ compound, which is absent during hydrogen reduction. Direct transformation of the sub-stoichiometric structures into Ni/La_2_O_3_ was observed.^9^

Already this example highlights the importance of strictly following the *in situ* evolution of crystalline structures for a full appreciation of structure–activity correlations. This refers to the formation of intermediate structures, but also to the crystalline monoclinic La_2_O_2_CO_3_ structure, which is most crucial for the necessary reversible CO_2_ activation/release cycle. Thanks to the crystalline nature of the participating structures, the bifunctionally operating Ni/La_2_O_2_CO_3_ interface, providing both methane activation (metallic Ni) and CO_2_ activation (carbonation of La_2_O_3_ to monoclinic La_2_O_2_CO_3_), can be directly visualized and studied. Even more, we were able to show the exclusiveness of the presence of the monoclinic La_2_O_2_CO_3_ structure with respect to enhanced DRM activity.

### 2.2. *In situ* activation of methanol steam reforming catalysts: Cu_51_Zr_14_

**Fig. 2:**
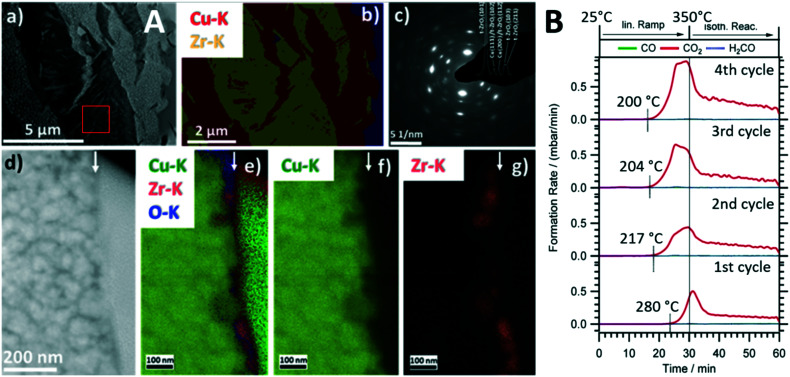
Structural and catalytic characterization of the *in situ* decomposition of crystalline Cu_51_Zr_14_ during methanol steam reforming operation. Panel A: Electron microscopy analysis of the decomposed state after the first catalytic cycle. Subpanel a): Overview high-angle annular dark-field (HAADF) image. Subpanel b): EDX analysis based on the Cu–K and Zr–K intensities of the same region. Subpanel c): Selected area electron diffraction (SAED) pattern of the region shown in subpanel a) marked by a red square. Subpanel d): HAADF image of the Cu-tetragonal ZrO_2_ interface (the surface is marked by a white arrow). Subpanels e)–g): EDX analysis of the same region. The superimposed Cu–K, Zr–K and O–K intensities are shown in e); the individual maps of the Cu–K and Zr–K intensities are shown in f) and g). The green intensity on the right part of subpanel e) refers to Pt used in the FIB cutting process. Panel B: Four consecutive catalytic methanol steam reforming profiles using crystalline Cu_51_Zr_14_. Reaction conditions detailed in ref. 8. Reproduced with permission from ref. 8, Wiley-VCH, 2021.

Intermetallic compounds have recently evolved as a promising materials class with a wide range of application possibilities in catalytic research.^18–21^ Although stable intermetallic compounds are used directly as catalyst materials, they are increasingly used as catalyst precursor structures, where the active species is formed through an activation step. The concept itself is very much related to the perovskite activation^9,10^ in a sense that the well-defined intermetallic compound starting structure paves the way to structurally steer the decomposition towards a metal–oxide composite with superior catalytic properties.^8^ We have shown the capabilities of this approach for a number of intermetallic compounds in a variety of reactions,^22^ but exemplify it for Cu_51_Zr_14_ in the methanol steam reforming reaction.^8^ Cu-containing compounds, with a technically used Cu/ZnO/Al_2_O_3_ system and ZrO_2_-doped composites as particular promising stable catalysts, represent the state-of-the-art.^23,24^ Materials development for Cu compounds in methanol steam reforming is essentially directed towards improving the poor sintering stability of Cu particles at elevated temperatures.^24^ Zr-based Cu intermetallic compounds represent a material class, where this drawback is circumvented by *in situ* activation and stabilization of the Cu particles using an extended ZrO_2_ matrix *in situ* formed by activation.^8,25,26^ Cu_51_Zr_14_ stands out particularly in terms of yielding a highly active MSR composite material after *in situ* decomposition. As shown in Fig. 2A, the crystalline Cu_51_Z_14_ structure is *in situ* decomposed during methanol steam reforming exclusively into a crystalline Cu/tetragonal ZrO_2_ composite. This is insofar remarkable as Cu is known to stabilize the tetragonal ZrO_2_ structure, which in contact to Cu yields a particularly CO_2_-selective methanol steam reforming catalyst,^27^ even activating itself during four consecutive methanol steam reforming cycles (Fig. 2B). The particular importance of a well-defined crystalline starting Cu_51_Zr_14_ compound is reflected in the close match of the cubic Cu and the tetragonal ZrO_2_ lattice constants, providing energy gain by epitaxial stabilization of the Cu/tetragonal ZrO_2_ interface.^25^ As for the perovskite case study discussed in section 2.1., the bifunctional operating mechanism for methanol steam reforming through methanol activation on metallic Cu and water activation on ZrO_2_ and/or at special crystalline interfacial sites is evident.^8^ Although the decomposition of such intermetallic compounds especially during methanol steam reforming has been known for some time,^8,22^ it is exactly the crystalline nature of the Cu_51_Zr_14_ compound that allows the transition of the precursor structure into the active phase by, *e.g.*, *in situ* X-ray diffraction correlated to catalytic selectivity and activity to be monitored.

### 2.3. Stability of oxycarbides: ZrO_0.31_C_0.69_

**Fig. 3:**
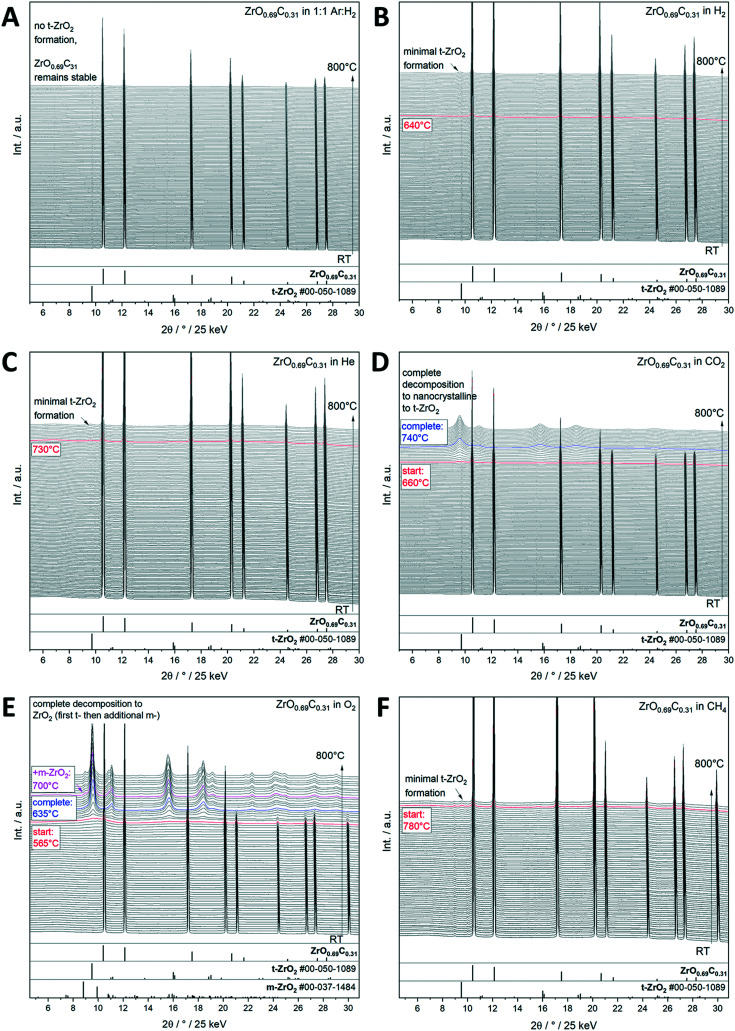
Decomposition study of crystalline ZrO_0.69_C_0.31_ in selected inert, oxidative and reductive gas atmospheres up to 800 °C as monitored by synchrotron-based *in situ* X-ray diffraction. Panel A: Ar : H_2_ mixture = 1 : 1, panel B: pure H_2_, panel C: pure He, panel D: pure CO_2_, panel E: pure O_2_, and panel F: pure CH_4_. The X-ray diffraction patterns are shown as waterfall plots. The temperatures where important structural changes are observed are marked accordingly. Reference patterns are indicated as bars below the diffractograms. In part reproduced with permission from ref. 12 Wiley-VCH, 2021.

Interstitial alloys of transition metals, mostly known as metal carbides and nitrides, have been extensively studied because of their promising physico-chemical properties differing from the parent metals.^28–30^ Interstitial incorporation of carbon into the lattice of group 4–6 transition metals modifies both their structural^29,31^ and electronic properties. Their associated chemical activity^29,30,33^ is similar to or sometimes even exceeds that of Pt-group metals.^32^ Transition metal carbides (TMCs) are therefore suitable catalysts for reforming processes, *e.g.*, hydrogenation of CO and the water gas shift (WGS) reaction.^34^ Important for electrochemical applications, the electrochemical stability of such transition metal carbides over wide potential and pH ranges is important for their use as low cost electrocatalysts in electrochemical reactions.^35,36^ The recurrent structural theme of most of those materials is the intrinsic propensity to be unstable towards oxycarbide or even oxide formation due to their high affinity to oxygen.^37–39^ In fact, it has been shown for many examples, including the most well-studied TiO_*x*_C_*y*_ and MoO_*x*_C_*y*_ materials,^37–40^ that the active phase of such transition metal carbides is an oxy-carbide structure and that the respective carbide under reaction conditions is an idealized benchmark state.^40^ Having said that, assessing the stability limits under realistic gas and liquid phase (electro-)catalytic conditions is of paramount importance for the use and projection of the performance of similar structures along the material axis. We exemplify this issue on a recently found novel ZrO_0.69_C_0.31_ oxy-carbide material,^41^ which has shown great potential for energy conversion reactions under strongly anodic conditions, *e.g.*, for alcohol or CO oxidation.^12^ To test the stability limits of crystalline ZrO_0.69_C_0.31_ under different gas atmospheres, *in situ* X-ray diffractograms collected from room temperature to 800 °C show that the oxycarbide material is stable under reductive conditions in both pure and Ar diluted H_2_ (Fig. 3A and B), as well as in He and CH_4_ (Fig. 3C and F), with only small amounts of tetragonal ZrO_2_ formed at high temperatures. In contrast, it is readily oxidized in O_2_ and CO_2_ with complete decomposition of ZrO_0.69_C_0.31_ into ZrO_2_ (Fig. 3D and E). Metallic conductivity of ZrO_0.69_C_0.31_ is preserved if minor amounts of ZrO_2_ are formed. In the case of complete decomposition, a pronounced increase of the impedance is observed, making the decomposition product an insulating material. The importance of the presence of a crystalline material is reflected in the fact that essentially only tetragonal ZrO_2_ as a decomposition product, but only in tiny amounts of the ambient-stable monoclinic modification, could be verified by *in situ* X-ray diffraction. This observation provides the link to the Cu/ZrO_2_ case study discussed in section 2.2., as we might infer that for all catalytic applications, either ZrO_0.69_C_0.31_ (if stable), tetragonal ZrO_2_ alone (in the case of full decomposition) or a Zr oxy-carbide/tetragonal ZrO_2_ interface (in the case of partial decomposition) is prevalent. As for Cu/ZrO_2_, the crystallinity of ZrO_0.69_C_0.31_ favors inferring the epitaxial stabilization effects between the tetragonal ZrO_0.69_C_0.31_ and tetragonal ZrO_2_ structures steering the decomposition towards tetragonal ZrO_2_.

### 2.4. Gas phase dependency of the polymorphic ZrO_2_ structure transformation

The gas-phase dependence of the crystalline tetragonal-to-monoclinic ZrO_2_ polymorphic transformation in a nutshell provides the great recurrent theme governing the structural and catalytic properties of the ZrO_2_-containing materials discussed in the previous sections. It is the central single most important parameter in catalyst development and in the establishment of structure–property relationships, and it very much depends on the crystallinity of the materials which allows the associated structural changes to be monitored. The Zr–O phase diagram is complex and involves at least three different crystalline modifications: the ambient-stable monoclinic structure and two tetragonal and cubic metastable high-temperature structures.^5^ The latter two can be stabilized and recovered to room temperature either through external doping, *e.g.*, with Y (yielding an yttrium-stabilized ZrO_2_ structure used, *e.g.*, as electrolyte and ion conductor in solid oxide fuel cells), *via* particle size control or *via* intrinsic “doping” by oxygen vacancies. It is well-known, that tetragonal ZrO_2_ can be stabilized by oxygen vacancies, and any application of tetragonal ZrO_2_ relies on the persistence of such vacancies during use to suppress the inevitable structure transformation to monoclinic ZrO_2_.^5^ As discussed in previous sections, the monoclinic bulk structure appears to be of inferior importance with respect to its use as part of a catalyst material in catalytic experiments, and it is mostly its surface chemistry that dictates its use, *e.g.*, in Cu/ZrO_2_ catalysts for methanol steam reforming.^24^ In contrast, tetragonal ZrO_2_ stands out as bulk crystalline structure and in many of its applications the crystalline bulk properties play an important part in explaining the catalytic reactivity pattern.^42^ The close match of the Cu and tetragonal ZrO_2_ lattice constants is one of the most prominent examples, as discussed in sections 2.2. and 2.3.^25^ It is therefore of utter importance to acquire knowledge about the gas-phase dependence of the tetragonal-to-monoclinic ZrO_2_ structure transformation, although the mechanistic ideas of the transition have been known for several decades. It is the so called martensitic structure transformation, which is athermic and diffusionless and propagates along the cracks of the structure resulting from the action of shear forces.^43^

**Fig. 4:**
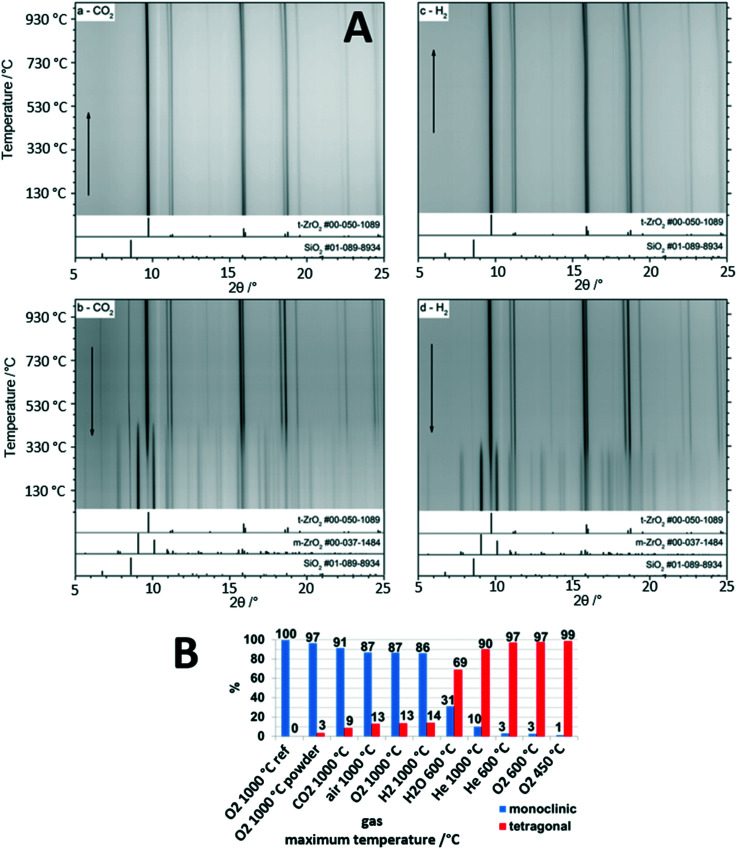
*In situ* X-ray diffraction studies of the evolution of crystalline bulk tetragonal ZrO_2_. Panel A: Temperature-dependence of the tetragonal-to-monoclinic ZrO_2_ structure transformation upon heating and re-cooling in H_2_ and CO_2_ from room temperature to 1000 °C and back. Panel B: Summary of the weight fraction analysis based on Rietveld refinement of tetragonal and monoclinic ZrO_2_ after treatments in selected gas atmospheres as indicated. Reproduced in part with permission from ref. 5, Royal Society of Chemistry, 2021.

As indicated in Fig. 4, the influence of the gas atmospheres on the extent of structure transition and therefore the individual weight fractions of still remaining tetragonal ZrO_2_ and gas-phase induced monoclinic ZrO_2_ is tremendous. The starting point of each experiment is structure-pure, well crystallized tetragonal ZrO_2_. In previous studies we have demonstrated the extraordinary stability of tetragonal ZrO_2_ up to 450 °C, essentially being independent of the treatment conditions.^42^ This also encompasses dry and moist conditions, which is insofar of importance as it allows the use of either structurally stable bulk structure-pure tetragonal ZrO_2_ or a crystallized metal (mostly Cu)/tetragonal ZrO_2_ interface for steam reforming reactions up to typical temperatures of around 400 °C. The astonishing suppression of the transformation to monoclinic ZrO_2_ is ascribed to the synthesis conditions, which involved water-free precipitation from a Zr propoxide precursor.^42^ Using this approach, a high number of bulk oxygen defects are retained within the tetragonal ZrO_2_ structure, which serve as the blockage to the polymorphic transformation and provide extra bulk structural stability.^5^ In due course, as shown in Fig. 4, we tried to deliberately trigger the structure transition by heating tetragonal ZrO_2_ in selected inert, reductive and oxidative gas environments. Exemplarily highlighted in Fig. 4A, the temperature dependence of the *in situ* collected X-ray diffractograms are shown under mildly reducing conditions in flowing CO_2_ (1 bar) and strongly reducing conditions in pure hydrogen (1 bar). The high stability of the crystalline tetragonal ZrO_2_ structure in both atmospheres up to 950 °C is immediately obvious. The formation of monoclinic ZrO_2_ is only observed upon re-cooling below around 700 °C in CO_2_ and 600 °C in H_2_. The onset temperature of the polymorphic transformation is higher (*i.e.*, earlier upon cooling) in CO_2_, and the amount of monoclinic ZrO_2_ in the composition mixture upon re-cooling to room temperature is lower in H_2_. We ascribed the higher transformation temperature in CO_2_ to a larger crystallite size of tetragonal ZrO_2_ (making it more unstable towards the structure transformation) and the higher amount of tetragonal ZrO_2_ in the final phase mixture in H_2_ to a better stabilization of bulk oxygen vacancies and the suppression of the replenishment of the latter. Fig. 4B summarizes the main features of the gas phase dependence: the amount of persisting tetragonal ZrO_2_ in the final mixture is higher the more reducing the gas atmosphere is, tetragonal ZrO_2_ is more stable the lower the temperature is, and moist conditions, even at a comparably low temperature of 873 K, trigger the formation of a considerable amount of monoclinic ZrO_2_. These findings are of special importance for water-containing reactions, such as methanol steam reforming, as the initially more oxidizing and moist conditions (getting progressively more reducing as the reaction proceeds by forming more hydrogen in the gas outlet) tend to favor the structure transformation. It should be noted that the deduction of these principles is only possible by closely monitoring the bulk structure and reactivity of crystalline tetragonal ZrO_2_. This is insofar important as in many studies with initially tetragonal ZrO_2_, once the reaction proceeds at elevated temperatures, a structural mixture of tetragonal and monoclinic ZrO_2_ is rather present.^5,42^

### 2.5. Carbon reactivity

**Fig. 5:**
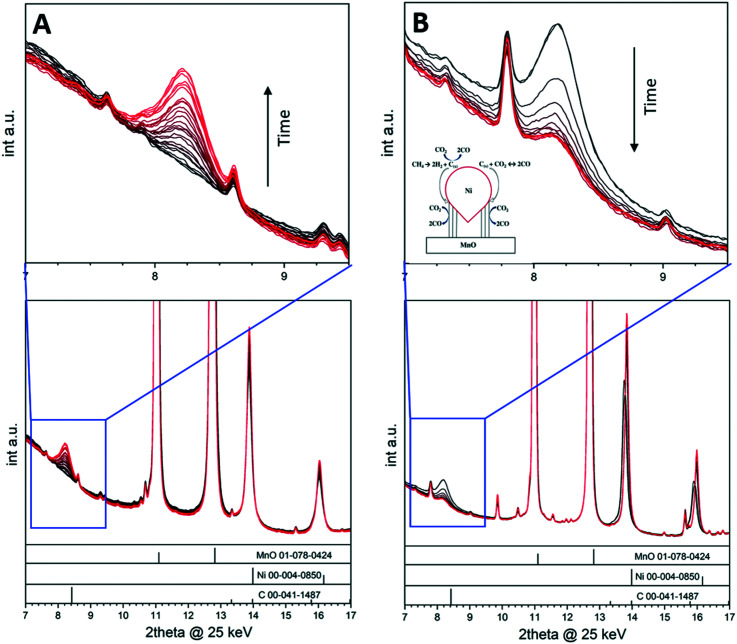
*In situ* collected X-ray diffraction patterns on 5% Ni/MnO. Panel A: Reaction temperature 600 °C, reactant ratio: CO_2_ : CH_4_ = 1 : 1, reaction time: 150 min. Panel B: Reaction temperature 800 °C, reactant ratio: CO_2_ : CH_4_ = 2 : 1, reaction time: 75 min. A pattern has been collected every 5 min. The diffractogram regions marked by the blue squares are enlarged at the top of each panel and display the evolution of the (002) graphite reflection as a function of time. Reference patterns are indicated as bars at the bottom of each panel. Reproduced with permission from ref. 11, Copyright American Chemical Society, 2021.

The deposition of carbon leading to catalyst deactivation is a common phenomenon in various reactions, and much research is put into assessing the carbon reactivity and clean-off mechanisms.^44–47^ Although carbon deposition is mainly viewed as a nuisance, carbon nevertheless can act as a crucial intermediate in catalytic reactions^48^ and in some cases even contribute beneficially to the overall catalytic properties.^11,48^ By focusing on a selected example in the dry reforming of methane reaction, we show how the monitoring of crystalline carbon species provides insight into the catalytic action of a Ni/MnO oxide interface during DRM operation.^11^ The dry reforming of methane reaction is best suited as an illustrative example in this respect, as the reaction of carbon following methane activation is part of the reversible carbon dioxide activation and release cycle.^49^ For La-based Ni catalysts, it is accepted that carbon dioxide activation involves the formation of an La-oxycarbonate that is decomposed through La_2_O_2_CO_3_(s) + C(s) → La_2_O_3_(s) + 2CO(g).^9^ The reactivity of the carbon intermediate is therefore crucial in the syngas production. Crystalline surface carbon can be formed *via* the Boudouard equilibrium, *via* direct decomposition of methane or *via* the reverse coal gasification process and, as such, is strongly individually dependent on the reaction conditions and structural and/or chemical properties of the catalyst material (*e.g.*, particle sizes and morphology or doping level).^11^ A 5% Ni/MnO catalyst has been suggested to be particularly stable at low temperatures, exhibiting small MnO-interface stabilized Ni particles and a quite low propensity for surface carbon formation.^49,50^ Ni particle sintering was offered as an explanation for deactivation at higher reaction temperatures. This catalyst material is therefore the ideal choice to decouple the effect of Ni particle sintering and the potential deactivation by the appearance of surface carbon, which is particularly aided by the appearance of crystalline graphite reflections that can be monitored during time-on-stream (Fig. 5). To illustrate the importance of *in situ* monitoring the carbon reactivity during the dry reforming reaction, we used the 5% Ni/MnO catalyst at different temperatures and reactant partial pressures. Changing the reactant ratio with time-on-stream results in a decrease in the deactivation rate of the catalyst, which can be correlated with the graphitic carbon growth and metal particle sintering as observed by *in situ* X-ray diffraction under DRM reaction conditions. We have shown that methane and carbon monoxide both separately yield graphitic surface carbon in the form of multiwalled carbon nanotubes, which can be oxidized by carbon dioxide. The correlation of catalytic experiments and *in situ* X-ray diffraction suggests that surface carbon acts as an intermediate in the carbon monoxide formation and that catalyst deactivation rather happens *via* metallic particle sintering. Fig. 5 shows a collection of *in situ* X-ray diffractograms and indicates the build-up of crystalline graphitic surface carbon at 600 °C during the measurement time of 150 min (highlighted region in the blue frame) in a 1 : 1 CO_2_ : CH_4_ mixture. Increasing the reactant ratio to CO_2_ : CH_4_ = 2 : 1 stopped the growth of carbon immediately and the amount of graphite remained constant for 75 min (not shown). Increasing the reaction temperature and observing the carbon reactivity at 800 °C (Fig. 5B) led to a swift decrease in the amount of carbon due to the thermodynamic feasibility of the coal gasification process in that particular temperature region. The schematic representation shown as inset in Fig. 5B summarizes the reactions at the Ni–MnO interface.

## 3. Summary and outlook

The presented Highlight article summarized in five selected examples the importance of the occurrence and reactivity monitoring of crystalline species and compounds to derive structure–property relationships in heterogeneous catalysis. Despite being a bulk-related phenomenon, the tracking of crystalline structures during (self-)activation is indispensable to disentangle the mechanism of complex reactions such as methanol steam or methane dry reforming. There is a rich pool of ways in which crystalline species can contribute to the understanding of catalytic action of different catalyst materials, which underscores the widespread occurrence and validation of the concept. Simple binary oxides, complex oxides such as perovskites or Ruddlesden–Popper structures, intermetallic compounds and alloys, and (oxy-)carbides or metal–oxide composites during catalyst activation or reaction are all affected distinctly and differently. This includes, but is not limited to

• catalyst bulk reconstruction (*e.g.*, the tetragonal-to-monoclinic ZrO_2_ polymorphic transformation),

• formation of transient structures (*e.g.*, the observation of La_2_NiO_4_*en route* from LaNiO_3_ to Ni/La_2_O_3_/La_2_O_2_CO_3_),

• catalyst self-activation (*e.g.* decomposition of Cu_51_Zr_14_ during methanol steam reforming),

• epitaxial stabilization effects (*e.g.*, the formation of Cu/tetragonal ZrO_2_*via* decomposition of Cu_51_Zr_14_),

• stability issues (*e.g.*, the reversible decomposition and re-formation of the oxycarbide electro-catalyst ZrO_0.31_C_0.69_ into ZrC and ZrO_2_ under oxidative and reductive atmospheres) and

• reactivity of crucial reaction intermediates (*e.g.*, formation of La-oxycarbonate structures through carbon dioxide activation during the methane dry reforming of LaNiO_3_ or formation of reactive carbon).

Despite the importance of the bulk crystalline species for the understanding of heterogeneous catalysis, the concept is only as strong as the availability of *in situ* methods that allow the monitoring of the respective species and the input from overlapping research areas all contributing to the bigger picture. In the present case, inorganic chemistry and crystal engineering (*via* providing preparation pathways to the relevant crystalline samples), crystallography (providing the structural input) and materials science (catalyst characterization *via* sophisticated *in situ* characterization) work together to derive a clear picture of the catalytic properties and the establishment of structure–property relationships. Insight into the development of crystalline structures is also given by complementary *in situ* imaging techniques, such as electron microscopy at high temperatures, which has seen tremendous advances recently especially in perovskite-related stability studies.^51,52^

## Conflicts of interest

There are no conflicts to declare.

## Supplementary Material
